# Adipose Tissue SIRT1 Regulates Insulin Sensitizing and Anti-Inflammatory Effects of Berberine

**DOI:** 10.3389/fphar.2020.591227

**Published:** 2020-12-17

**Authors:** Yun Shan, Shuchen Zhang, Bin Gao, Shu Liang, Hao Zhang, Xizhong Yu, Juan Zhao, Lifang Ye, Qin Yang, Wenbin Shang

**Affiliations:** ^1^ Department of Endocrinology, Jiangsu Province Hospital of Chinese Medicine, The Affiliated Hospital of Nanjing University of Chinese Medicine, Nanjing, China; ^2^ Key Laboratory for Metabolic Diseases in Chinese Medicine, First College of Clinical Medicine, Nanjing University of Chinese Medicine, Nanjing, China; ^3^ School of Medicine and Life Sciences, Nanjing University of Chinese Medicine, Nanjing, China; ^4^ Department of Medicine and Physiology, UC Irvine Diabetes Center, Center for Epigenetics and Metabolism, University of California at Irvine, Irvine, CA, United States

**Keywords:** berberine, SIRT1, insulin resistance, inflammation, adipose tissue, acetylation

## Abstract

Berberine (BBR), which is an active component of Coptis chinensis Franch, has been reported to improve glucose metabolism and insulin resistance in animal and human studies, predominantly via activation of the 5′-adenosine monophosphate kinase (AMPK) pathway and suppression of the inflammation response. However, the mechanisms underlying the effects of BBR on AMPK and inflammation remain unclear. In this present study, we found that BBR upregulated SIRT1 expression in 3T3L-1 adipocytes and adipose tissue. Inhibition of SIRT1 blunted the BBR-induced increase in glucose consumption and uptake in adipocytes. The BBR-induced activation of the AMPK pathway and AKT phosphorylation in adipocytes and adipose tissue were also attenuated by inhibition or knockout of *Sirt1*. The BBR-induced improvement of systemic insulin sensitivity was impaired by *Sirt1* knockout in HFD-induced obese mice. The suppressing effects of BBR on systemic and local inflammatory responses, such as serum concentrations and expression of inflammatory cytokines, phosphorylation of c-Jun N-terminal kinase (JNK) and IKKβ, and the accumulation of F4/80-positive macrophages in adipose tissue were also attenuated in *Sirt1* knockout mice. The BBR-induced decrease in PGC-1α acetylation was reversed by inhibition or knockout of *Sirt1* in adipocytes and adipose tissue. Together, these results indicate that adipose tissue SIRT1 is a key regulator of the insulin sensitizing and anti-inflammatory effects of BBR, which contributes to the improvement of metabolic dysregulation.

## Introduction

Insulin resistance is a major factor involved in obesity-related clinical disorders, such as glucose intolerance, type 2 diabetes, dyslipidemia, hypertension, and cardiovascular disease (Moller and Kaufman, 2005). Chronic, low-grade inflammation in white adipose tissue plays a critical role in the development of obesity-associated local and systemic insulin resistance, and presents as the overproduction of proinflammatory cytokines and accumulation of macrophages in the adipose tissue of obese patients (Johnson and Olefsky, 2013; McLaughlin et al., 2017). A decrease in 5′-adenosine monophosphate kinase (AMPK) activity in adipose tissue also contributes to the pathogenesis of insulin resistance, and pharmacological AMPK activators may be used for ameliorating insulin resistance in type 2 diabetes (Ruderman et al., 2013).

Berberine (BBR) is an active component of *Coptis chinensis* Franch and has long history of use in Chinese medicine (Meng et al., 2018). Various pharmacological and therapeutic effects of BBR have been reported in recent years (Imenshahidi and Hosseinzadeh, 2016), and the antidiabetic properties of BBR have been demonstrated in several human and animal studies (Chang et al., 2015; Imenshahidi and Hosseinzadeh, 2016; Belwal et al., 2020). BBR appears to exert its insulin sensitizing activity via activation of the AMPK pathway and its anti-inflammatory properties (Li et al., 2014; Chang et al., 2015; Neag et al., 2018). BBR improves glucose and lipid dysregulation by activating AMPK in adipocytes, myotubes, hepatocytes, and cardiomyocytes (Lee et al., 2006; Zhou et al., 2007; Yin et al., 2008; Kim et al., 2009; Chang et al., 2013). Moreover, BBR-induced inhibition of the inflammatory response in adipose tissue leads to improvement of insulin resistance. We found that BBR reduced serum levels of tumor necrosis factor-α (TNF-α) and interleukin 6 (IL-6), and inhibited activation of inhibitor of κB kinase beta (IKK-β), c-Jun N-terminal kinase (JNK), and proinflammatory M1 macrophages in the adipose tissue of high-fat diet (HFD)-induced obese mice, with concurrent improvements in glucose tolerance and insulin sensitivity (Shang et al., 2010; Ye et al., 2016). It was previously reported that BBR treatment significantly downregulated the expression of proinflammatory genes, such as TNF-α, IL-1β, and IL-6 and monocyte chemoattractant protein-1 (MCP-1), in the adipose tissue of obese db/db mice and inhibited lipopolysaccharide (LPS)-induced expression of proinflammatory genes, including IL-1β, IL-6, iNOS, and MCP-1, as well as proinflammatory signals in macrophages dependent on AMPK activation (Jeong et al., 2009). However, the direct mechanisms involved in BBR’s effects on AMPK and inflammation are not well understood.

(1) There is growing evidence to suggest that silent information regulator 1 (Sirt1) plays a role in glucose homeostasis and insulin sensitivity via its deacetylase activity (Liang et al., 2009; Cao et al., 2016). Overexpression of Sirt1 and various Sirt1 activators stimulate AMPK signaling, leading to improved insulin resistance in adipocytes and muscle tissue (Price et al., 2012; Chen et al., 2018). On the other hand, Sirt1 modulates insulin sensitivity by inhibiting inflammation in adipocytes and macrophages and altering macrophage recruitment in adipose tissues (Yoshizaki et al., 2009; Yoshizaki et al., 2010; Ka et al., 2015; Hui et al., 2017). BBR and SIRT1 show striking similarities in the regulation of insulin sensitivity involving the AMPK pathway and inflammatory response in adipose tissue, suggesting that BBR may depend on the Sirt1 pathway to improve insulin resistance.
(2) We recently reported that BBR suppressed the inflammatory responses in macrophages through inhibition of the nuclear factor kappa-light-chain-enhancer of activated B cells (NF-κB) signaling via SIRT1-dependent mechanisms (Zhang et al., 2017). Other studies have also indicated that BBR inhibits lipid accumulation in hepatocytes and rescues mitochondrial function in muscle in a SIRT1-dependent manner (Gomes et al., 2012; Sun et al., 2018). Therefore, we hypothesized that adipose tissue SIRT1 mediates BBR-induced activation of AMPK and suppression of inflammation. The present study tested this hypothesis in 3T3-L1 adipocytes and adipose tissue from HFD-induced obese mice. We found that BBR upregulated SIRT1 expression both in 3T3-L1 adipocytes and mouse adipose tissue. BBR-induced activation of the AMPK pathway and suppression of the inflammatory response were attenuated by SIRT1 inhibition in 3T3-L1 adipocytes or *Sirt1* knockout in obese mice. Furthermore, SIRT1 mediated the alleviating effect of BBR on insulin sensitivity in adipose tissue and the whole body, leading to the amelioration of metabolic dysregulation.

## Materials and Methods

### Chemicals and Reagents

Berberine (≥98% purity) and EX-527 were purchased from Sigma (Saint Louis, MO). Dulbecco’s modified Eagle’s medium (DMEM), fetal bovine serum (FBS), and recombinant mouse tumor necrosis factor-α (TNF-α) were purchased from Gibco (Grand Island, NY). Recombinant human insulin was purchased from Lilly (Fegersheim, France). Antibodies against Sirt1, JNK, p-IKKβ, IKKβ, p-Akt (Ser473), Akt, AMPK-α, p-AMPK-α (Thr172), liver kinase B1 (LKB1), p-LKB1, acetyl-CoA carboxylase (ACC), p-ACC, acetylated-lysine antibody, anti-rabbit and anti-mouse horseradish peroxidase (HRP)-conjugated antibodies were obtained from Cell Signaling Technology (Beverly, MA). Antibodies against p-JNK, phospho-insulin receptor substrate (p-IRS^Tyr612^), IRS-1, and F4/80 were purchased from AbCam (Cambridge, MA). Antibodies against peroxisome proliferator-activated receptor gamma coactivator 1-alpha (PGC-1α) and β-actin were purchased from Santa Cruz Biotechnology (Santa Cruz, CA). All other chemicals and regents were purchased from Sigma (Saint Louis, MO) unless otherwise described.

### Cell Culture

3T3-L1 preadipocytes were obtained from ATCC and grown in DMEM supplemented with 10% FBS. The cells were induced to differentiate as previously described (Shang et al., 2008). Briefly, 2 days after reaching confluence, preadipocytes were induced to differentiate using DMEM supplemented with 10% FBS, 10 mg/ml insulin, 0.5 mM 3-isobutyl-1-methylxanthine, and 1 mM dexamethasone. After 2 days, the differentiation medium was replaced with DMEM containing 10% FBS and 10 mg/ml insulin. Two days later, the medium was changed to DMEM supplemented with 10% FBS. On day 10 after the initial induction, small lipid droplets were observed in 90% of the cells. The accumulation of lipid droplets was considered to be indicative of full differentiation, and these cells were used in the experiments. BBR was dissolved in dimethyl sulfoxide (DMSO) to create a 1000-fold stock, and added to the medium at various concentrations. DMSO was present in the control culture at a concentration 0.1% (v/v).

### Animal Experiments

Eight-week-old male C57BL/6J mice and male *Sirt1* heterozygous knockout (*Sirt1* ^+/−^) mice in a C57BL/6 background were purchased from the Animal Model Research Center of Nanjing University (Nanjing, China). The mice were housed with three mice per cage housed in a room maintained at 23 ± 1 °C with a 12 h light/dark cycle and free access to water and food. Mice with the same genotypes were randomized and fed ad libitum with either a standard laboratory chow diet containing approximately 10% fat (SLAC, Shanghai) or HFD containing 60% fat (Research Diets, USA) for 12 weeks. After 12 weeks, the mice were maintained on HFD and randomly assigned to receive oral administration of either vehicle (0.9% saline) or BBR at concentrations of 25 or 50 mg/kg body weight per day via gavage for 2 weeks (*n* = 6 per group). The mice were then sacrificed by decapitation. Blood was collected and the liver, epididymal fat tissues were dissected and immediately frozen in liquid nitrogen and stored at −80 °C. All animal studies were approved by the Animal Care and Use Committee at the University of Nanjing University of Chinese Medicine and were in accordance with the Regulation on the Administration of Laboratory Animals of China (2017 Revision).

### siRNA Silencing

Lentiviral shRNA expression vectors specific for *Sirt1* were purchased from Genepharma (Shanghai, China) with the following target sequence: 5′-CCG​UCU​CUG​UGU​CAC​AAA​UTT-3′. The following sequences were used for non-targeting control vector: 5′-TTC​TCC​GAA​CGT​GTC​ACG​T-3′. Mature 3T3-LI adipocytes were infected with the lentiviral vectors or non-targeting control vector at 1 × 10^8^ transducing units (TU)/mL for 24 h. The infection medium was replaced with culture medium containing 10% FBS and refreshed 48 h later. Reporter gene expression was examined by fluorescence microscopy. When the efficiency of infection exceeded 80%, cells were maintained in DMEM containing 0.2% bovine serum albumin (BSA) for 8–12 h for subsequent experiments. Knockdown of *Sirt1* expression was evaluated by Western blot analysis.

### Quantitative RT-PCR

Total RNA was isolated from 3T3-L1 adipocytes and epididymal adipose tissue from mice using Trizol reagent (TAKARA, Ohtsu, Japan). Reverse transcription was performed using SuperScript III Reverse transcriptase and Oligo (dT) primer (TAKARA, Ohtsu, Japan). PCR amplification was performed with SYBR Premix Ex TaII (TAKARA, Ohtsu, Japan) using the Applied Biosystems 7500 Real-Time PCR System. Fold changes in expression were analyzed using the 2^−ΔΔ^ Ct relative quantitative method. This Primers used in the study were included in Supplementary Tabel S1.

### Western Blot Analysis

Total protein from adipose tissue or adipocyte lysates were extracted using lysis buffer (50 mM Tris, 50 mM KCl, 20 mM NaF, 1 mM Na_3_VO_4_, 10 mM EDTA, 1% NP-40, 1 mM phenylmethylsulfonyl fluoride, and 5 g/ml leupeptin, pH 8.8). Protein concentration was determined using BCA protein assay kits (Pierce, Rockford, IL). SDS–PAGE was used to resolve 30 μg of protein, which was then transferred to PVDF membranes. The membranes were incubated using blocking buffer [5% (w/v) BSA powder in 1 × Tris-buffered saline containing 0.1% Tween-20] for 2 h, and incubated with a primary antibody overnight at 4 °C. The membranes were then washed and incubated for 2 h at room temperature with HRP-conjugated secondary antibodies. After washing, the ECL Plus system (GE Healthcare) was used to detect the protein signal. Immunoblots were developed using the ECL kit.

### Immunoprecipitation

Total protein from adipose tissue or adipocytes was extracted using lysis buffer. The lysate was then centrifuged and diluted with precooled lysis buffer (2 μg/μL). Next, 50 μL of Protein A/G PLUS-Agarose (Santa Cruz, CA) was added to 100 μL of preclear lysate. The supernatant was added to 2 μg of antibody or IgG and incubated overnight at 4 °C. Next, 50 μL of protein G-agarose was added to the mixture. The resins were then washed twice with a washing buffer and bound proteins were subjected to Western blot analysis.

### Glucose Consumption and Uptake

Glucose consumption was measured as previously described (Yin et al., 2008). Differentiated 3T3-L1 adipocytes were preincubated with DMEM containing 0.2% BSA for 12 h and the medium was removed. To measure glucose consumption, adipocytes were pretreated with 1 μM EX-527 or Lv-*Sirt1* then treated with phenol-free 0.2% BSA–DMEM with or without 5 μM BBR for 24 h, and glucose concentrations of DMEM were determined using the glucose oxidase method (Wako Pure Chemical Industries, Japan). Glucose consumption was calculated by subtracting the glucose concentration of the blank wells from that of the cell-plated wells. Glucose uptake was measured using the modified method as previously described (Zou et al., 2005). Briefly, adipocytes were pretreated with 1 μM EX-527 then treated with phenol-free 0.2% BSA–DMEM with 5 μM BBR in the presence of 1 ng/ml TNF-α for 24 h. The cells were then washed with phosphate-buffered saline (PBS) and then incubated in fresh glucose-free DMEM containing 1 nM insulin for 15 min. Next, the cells were treated with PBS containing 30 μM d-glucose analog 2-[ N-(7-nitrobenz-2-oxa-1,3-diazol-4-yl) amino]-2-deoxy-d-glucose (2-NBDG, Invitrogen, Madrid, Spain) for 2 h and 1 μg/ml DAPI for 10 min. The cells were then washed twice with PBS. Images were acquired using a High Content Screening Operetta System (Operetta, PerkinElmer, United States). Overall glucose uptake was calculated by quantifying the green fluorescence from four independent capturings of random fields.

### Glucose and Insulin Tolerance Tests

Glucose tolerance was measured in mice using oral glucose tolerance test (OGTT), after a 16-h overnight fast. Mice were administered 2 g/kg body weight of glucose via a gavage tube. Blood was collected from a tail vein at 0, 15, 30, 60, and 120 min, and blood glucose levels were measured using a OneTouch Ultra Blood Glucose Meter (Johnson & Johnson Medical, Shanghai, China).

Three days after performing the OGTT, mice were fasted overnight for 16 h and an insulin tolerance test (ITT) was performed using intraperitoneal injection of 1 IU/kg body weight of insulin (Lilly). Blood glucose was measured as described for the OGTT.

### Immunohistochemistry

Formalin-fixed paraffin-embedded adipose tissue sections were deparaffinized and rehydrated prior to incubation with an antigen unmasking solution. Sections were exposed to 3% H_2_O_2_ for 10 min. After washing with PBS, sections were incubated with serum diluted in PBS for 1 h, and then F4/80 antibody (1:200) was applied to adipose tissue. Slides were stored overnight at 4 °C in a humidified chamber. Sections were covered in an appropriate diluted biotinylated secondary antibody for 1 h at room temperature and then incubated with ABC reagent (Vector Laboratories, United States) for 30 min. Diaminobenzidine solution (0.05%) was applied to slides. Adipose tissue slices were visualized using a microscope.

### Liver Triglyceride Measurement and Oil Red O Staining

Liver tissues were placed in ethanolic KOH and mixed by vortex prior to incubation until the tissue was completely digested. The homogenates were then centrifuged at 10,000 rpm for 5 min and the supernatants transferred to new tube and 1 M MgCl_2_ was added. After centrifugation, the supernatants were collected to determine liver triglyceride levels using Serum Triglyceride Determination kits (Sigma, United States) and a microplate reader (PerkinElmer, Unites States). For histopathological evaluation, paraffin-embedded liver sections were sliced to a thickness of 4 μm and examined by hematoxylin and eosin (H&E) staining. Frozen liver sections were sliced to a thickness of 10 μm and examined by oil red O staining using averaged hepatocyte areas.

### Immunoassay

Serum concentrations of MCP-1, IL-6, and IFN-γ were quantified using Luminex Multiplex Assay kits (ebioscience, Vienna, Austria). Serum insulin and TNF-α were detected using a mouse ELISA kit (Mercodia, Uppsala, Sweden or Life technology, Frederick, MD) according to the manufacturer’s instructions.

### Statistical Analysis

Statistical analyses were performed using GraphPad PRISM 6.0. All values were expressed as mean ± SD. All data were from at least three independent experiments. Differences among multiple groups were determined using one-way analysis of variance followed by Tukey’s post-hoc test, with *p*-values <0.05 considered significant.

## Results

### Berberine Upregulated SIRT1 Expression in 3T3L-1 Adipocytes

**FIGURE 1:**
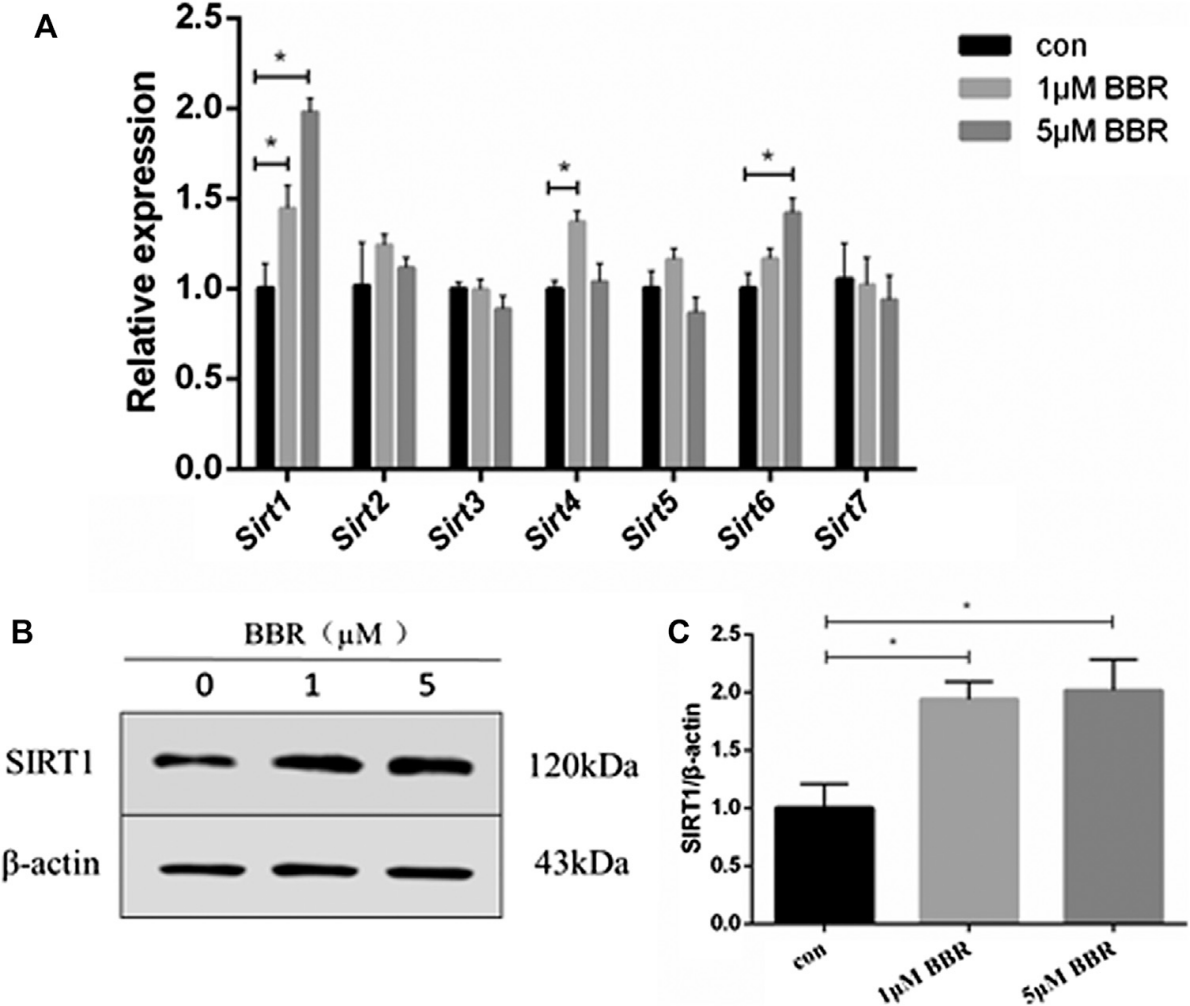
BBR upregulates SIRT1 expression in 3T3-L1 adipocytes. 3T3-L1 adipocytes were treated with BBR at various concentrations (1 and 5 μM) for 24 h. **(A)** Relative mRNA levels of *Sirt*1-7 in 3T3-L1 adipocytes measured by real-time PCR (*n* = 3). **(B)** Protein expression of SIRT1 in 3T3-L1 adipocytes was measured by Western blot analysis **(C)** Band intensities were quantified by normalizing to actin (*n* = 3). Quantitative data are presented as mean ± SD. *p < 0.05.

Differentiated 3T3-L1 adipocytes were treated with BBR at various concentrations (1 and 5 μM) for 24 h, and mRNA levels of Sirt 1–7 were measured. The results showed that BBR treatment increased the expression of Sirt 1, 4, and 6 significantly, in a dose-dependent manner (*p* < 0.05, Figure 1A). Our previous and other studies show that 5 μM BBR increases maximum glucose consumption in 3T3-L1 adipocytes without a significant decrease in the cell viability (Zhou et al., 2007; Yin et al., 2008). Therefore, 5 μM BBR was used in subsequent experiments in adipocytes. BBR treatment increased SIRT1 expression of in adipocytes at both the mRNA and protein levels.

### Inhibition of SIRT1 Attenuated the Berberine-Induced Increase of Glucose Consumption and Adenosine Monophosphate Kinase Phosphorylation in Adipocytes

**FIGURE 2:**
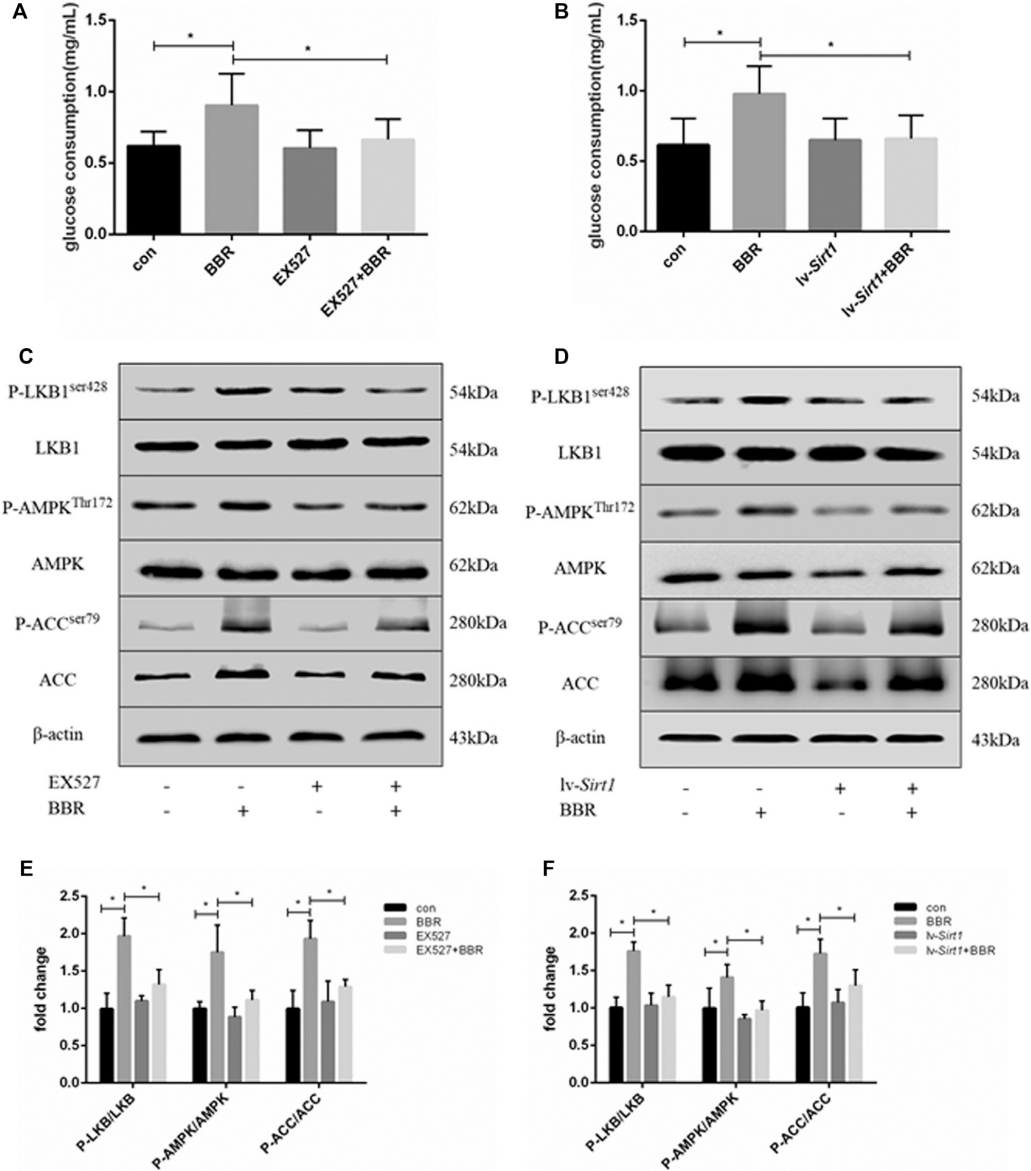
SIRT1 inhibition weakened the effect of BBR on the AMPK pathway and glucose consumption in adipocytes. 3T3-L1 adipocytes were pretreated with or without 1 μmol/L EX-527, or transfected with or without 1 × 10^8^ lentiviral shRNA expression vector as described in the Materials and Methods section and then treated with 5 μM BBR for 24 h **(A,B)** Glucose consumption was measured (*n* = 4). **(C–F)** Phosphorylation of LKB1, AMPK, and ACC was measured by Western blotting. Band intensities were quantified by normalizing to the corresponding total protein levels (*n* = 3–4). Quantitative data are presented as mean ± SD. *p < 0.05.

Previous studies showed that BBR can increase glucose consumption and uptake in adipocytes via activation of the AMPK pathway (Zhou et al., 2007). Therefore, we investigated whether the effect of BBR on glucose consumption and the AMPK pathway was dependent on SIRT1. First, we transduced adipocytes with lentiviral vectors expressing SIRT1 shRNA or nonsense shRNA. SIRT1 protein levels were reduced by the lentiviral vector for SIRT1 shRNA, the effect reached the maximum at 1 × 10^8^ TU/mL which was used in the following experiment. The non-targeting control did not affect the expression of SIRT1 (Supplementary Figure S1). As shown in Figures 2A,B, BBR treatment significantly increased glucose consumption in 3T3-L1 adipocytes (*p* < 0.05). However, inhibition of SIRT1 using the SIRT-specific chemical inhibitor, EX-527 (Figure 2A), or siRNA-mediated knockdown (Figure 2B) significantly suppressed the BBR-induced increase in glucose consumption (*p* < 0.05). Furthermore, the AMPK pathway was activated by BBR treatment, as indicated by the increased phosphorylation of LKB1, AMPK and ACC (Figures 2C,D), which was elevated significantly compared with that in the cells without BBR treatment (*p* < 0.05; Figures 2E,F). But the BBR-induced phosphorylation of LKB1, AMPK and ACC was attenuated by induced SIRT1 inhibition, which was induced by the treatment of EX-527 or lv-Sirt1 vector (*p* < 0.05; Figures 2C,E) or by (*p* < 0.05; Figures 2D,F). These results suggest that BBR activated the AMPK pathway to increase glucose metabolism in adipocytes, and was dependent on SIRT1.

### SIRT1 Inhibition Blunted the Sensitizing Insulin Action of Berberine in Adipocytes

**FIGURE 3:**
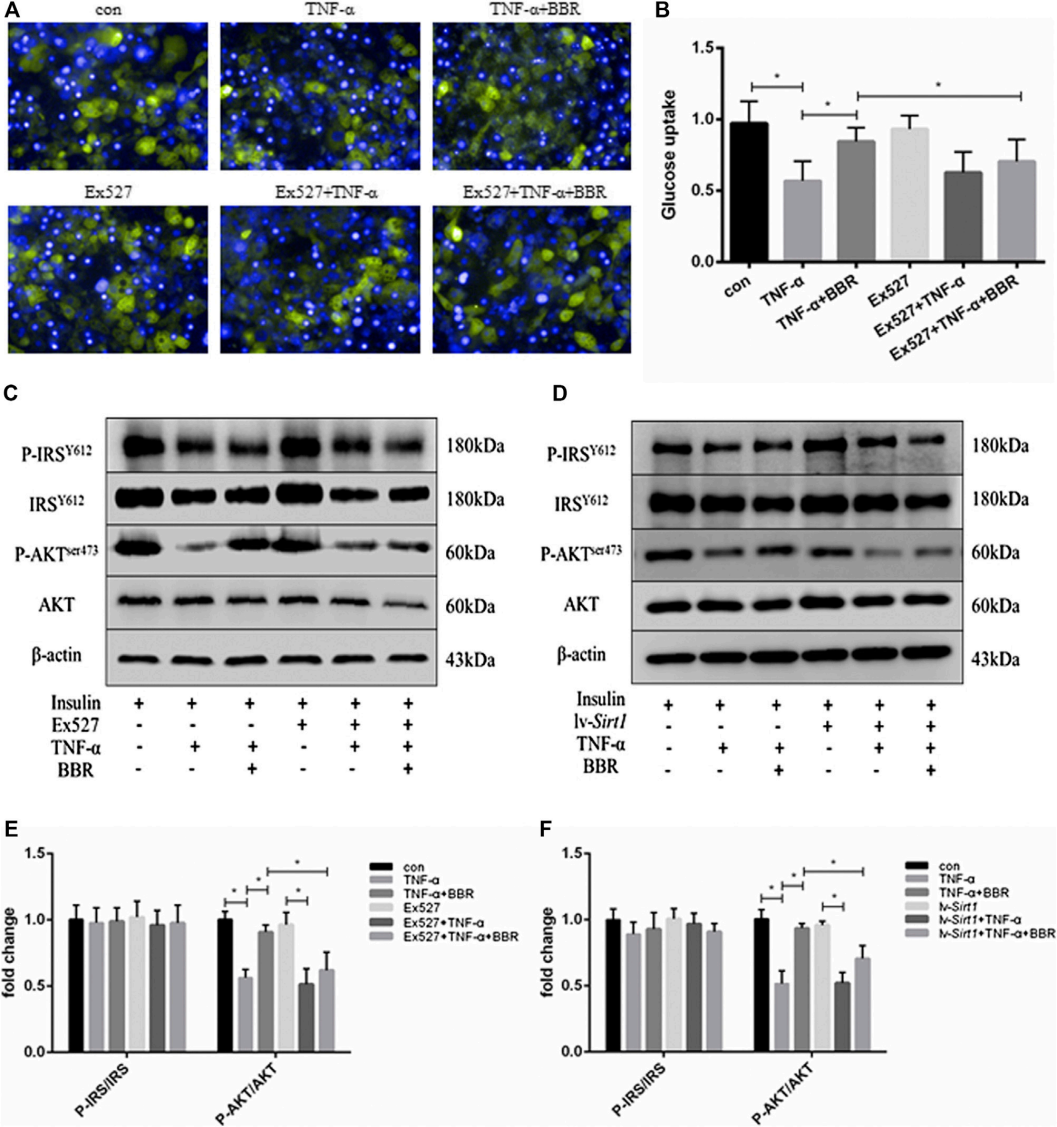
SIRT1 inhibition blunted the sensitizing insulin activity of BBR in adipocytes. 3T3-L1 adipocytes pretreated with EX-527 (1 μM) or transfected with or without 1 × 10^8^ lentiviral siRNA vector were treated with 5 μM BBR in presence of 1 ng/ml TNF-α for 24 h and then stimulated with 1 nM insulin for 15 min **(A,B)** Adipocytes were treated with 30 μM 2-NBDG for 2 h and 1 μg/ml DAPI for 10 min. Green fluorescence of glucose in adipocytes was recorded and quantified (*n* = 4). **(C,D)** Phosphorylation of IRS-1, AKT, and actin was detected by Western blotting **(E,F)** Band intensities of P-IRS-1and P-AKT were quantified by normalizing to the corresponding total protein levels (*n* = 3). Quantitative data are presented as mean ± SD. *p < 0.05.

BBR has been shown to restore the impaired insulin signaling pathway in insulin-resistant adipocytes (Zhou et al., 2007; Liu et al., 2010). In the present study, adipocytes were treated with TNF-α for 24 h to induce insulin resistance, which exhibited impaired insulin-stimulated glucose uptake (Figures 3A,B) and insulin signaling (Figures 3C,D). Insulin-stimulated glucose uptake in adipocytes was decreased by TNF-α treatment (*p* < 0.05; Figure 3B), but could be partly restored by BBR treatment (*p* < 0.05; Figure 3B). In the presence of TNF-α and insulin, the scale of the glucose uptake induced by BBR treatment in adipocytes pretreated with EX-527 was significantly lower than that in adipocytes without EX-527 treatment (*p* < 0.05; Figure 3B). Consistent with these result, insulin-stimulated phosphorylation of AKT^Ser473^ was suppressed by TNF-α treatment, and BBR treatment partly recovered the inhibited phosphorylation of AKT^Ser473^, but not phosphorylation of IRS-1^Y612^ (Figures 3C,D). EX-527 and SIRT1 siRNA weakened BBR’s ability to recover AKT phosphorylation (*p* < 0.05; Figures 3E,F). These results indicated that BBR improves the impaired insulin action induced by TNF-α in adipocytes via the SIRT1 pathway.

### Sirt1 Knockout Impaired Berberine-Induced Improvement of Systemic Insulin Sensitivity in HFD-Induced Obese Mice

**FIGURE 4:**
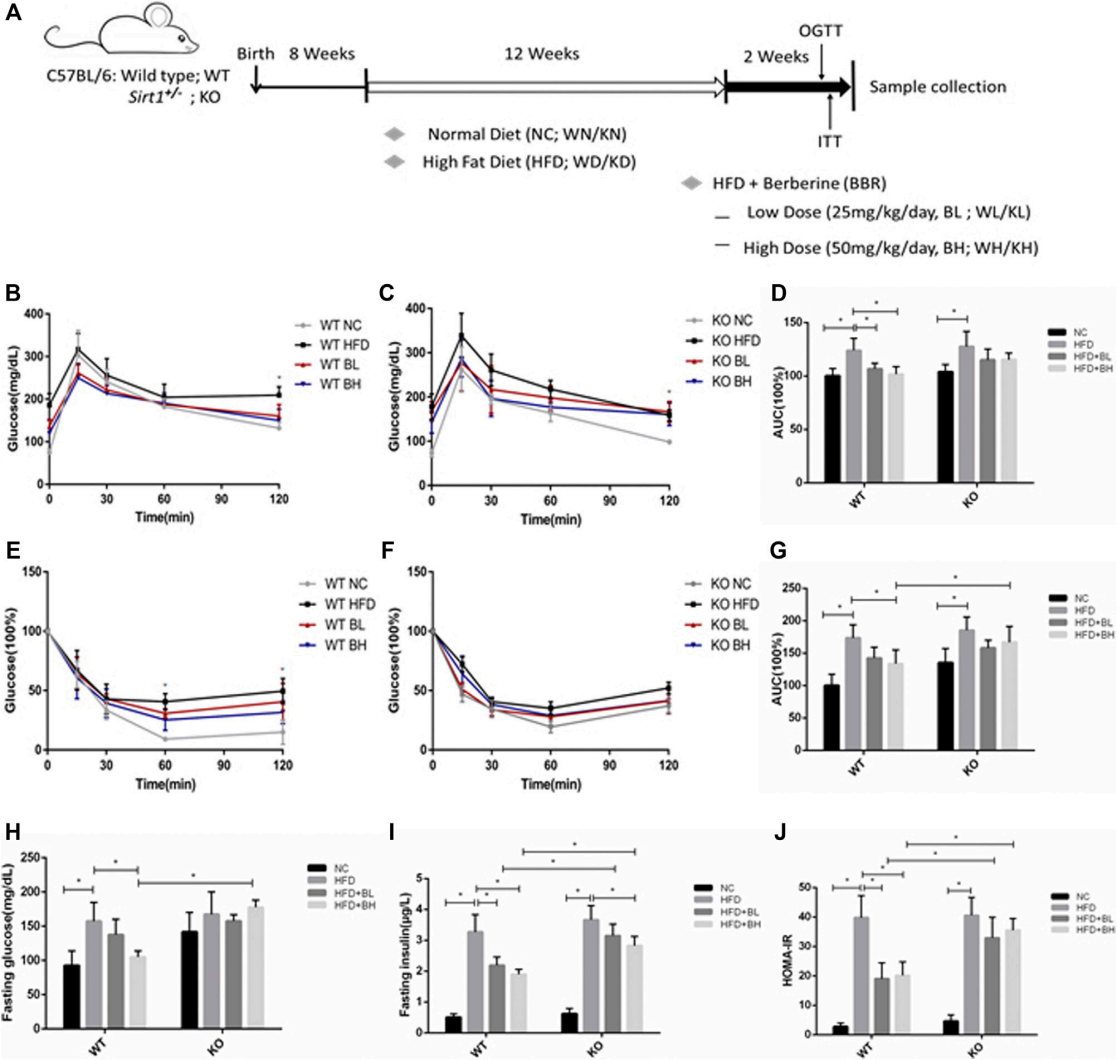
BBR’s action on systemic insulin sensitivity was impaired by SIRT1 knockout in HFD-induced obese mice. Wild Type (WT) or *Sirt* ^+/−^ (KO) C57BL/6 mice fed either normal chow (NC) or high-fat diet (HFD) were treated with 25 mg/kg (BL) or 50 mg/kg (BH) BBR for 2 weeks. **(A)** Schematic figure illustrating the animal experiment **(B,C)** Oral glucose tolerance test (OGTT). **(D)** Area under curve of OGTT **(E,F)** Insulin tolerance test (ITT). **(G)** Area under curve of ITT **(H)** Serum fasting glucose concentrations. **(E)** Serum fasting insulin concentrations **(J)** Homeostasis model assessment of insulin resistance (HOMA–IR) was calculated as the product of the fasting concentrations of plasma glucose (mmol/L) and plasma insulin (ng/L) divided by 22.5 (*n* = 6). Data are expressed as mean ± SD. ^*^ *p* < 0.05.

BBR has been shown to improve insulin resistance in mice and humans (Belwal et al., 2020). To further clarify whether the insulin sensitizing effect of BBR depends on SIRT1, we used *Sirt1* heterozygous knockout (*Sirt1* ^+/−^) mice (since SIRT1 homozygous knockout is embryonic lethal) to observe the role of SIRT1 on the effect of BBR on insulin sensitivity. Wild type (WT) and *Sirt1* ^+/−^ mice were fed either a normal chow (NC) or a HFD to induce obesity for 12 weeks and were then administered different doses of BBR (25 or 50 mg/kg body weight) for 2 weeks. In WT mice fed with HFD, both doses of BBR significantly (*p* < 0.05) improved glucose tolerance in the obese mice, as measured by OGTT (Figures 4B,D), and also significantly (*p* < 0.05) ameliorated insulin sensitivity, as measured by ITT (Figures 4E,G). Fasting glucose and insulin levels and homeostatic model assessment of insulin resistance (HOMA–IR) in BBR-treated mice were reduced significantly (*p* < 0.05; Figures 4H–J). However, in *Sirt1* ^+/−^ mice fed with HFD, BBR treatment did not significantly (*p* > 0.05) affect glucose tolerance, as measured by OGTT (Figures 4C,D), or insulin sensitivity (*p* > 0.05), as measured by ITT (Figures 4F,G), there was no significant difference between in HFD fed *Sirt1* ^*+/−*^ mice treated with BBR and vehicle. Knockout of *Sirt1* partly restored the BBR-induced decrease in fasting glucose (Figure 4H), as well as fasting insulin levels and HOMA–IR (Figures 4I,J), High dose of BBR also significantly reduced the body weight and epididymal fat weight (*p* > 0.05; Supplementary Figures S2A,D) in WT obese mice, without a decrease in food intake (Supplementary Figure S2C); however, this effect was not significant in *Sirt1* ^*+/−*^ obese mice (Supplementary Figures S2B,D). Furthermore, BBR significantly lowered serum levels of total cholesterol, high-density lipoprotein cholesterol, and low-density lipoprotein cholesterol in WT obese mice, but not in *Sirt1* ^*+/−*^ obese mice (Supplementary Figures S3B–D).

Obesity-related insulin resistance is also characteristic of hepatosteatosis. Therefore, liver dysfunction and hepatic lipid accumulation was evaluated in WT and *Sirt1* ^*+/−*^ obese mice treated with or without BBR. In WT obese mice, BBR decreased the liver weight and triglyceride content significantly (Supplementary Figures S4A,4B), consistent with a decrease in serum levels of aspartate transaminase (AST) and alanine aminotransferase (ALT) (Supplementary Figures S4C,D). A visible decrease in intracellular lipid droplet accumulation was also observed in BBR-treated WT HFD-fed mice liver sections stained with H&E (Supplementary Figure S4E) or O oil red (Supplementary Figure S4F). The lowering effects of BBR on liver weight, liver TG content, AST, and ALT were significantly weakened in *Sirt1* ^*+/−*^ obese mice (Supplementary Figures S4A–F).

These results demonstrated that BBR alleviated insulin resistance dependent of SIRT1 in HFD-induced obese mice, leading to beneficial metabolic effects.

### SIRT1 Was Required for Berberine Induction of Adenosine Monophosphate Kinase Activation and Insulin Signaling in Adipose Tissue

**FIGURE 5:**
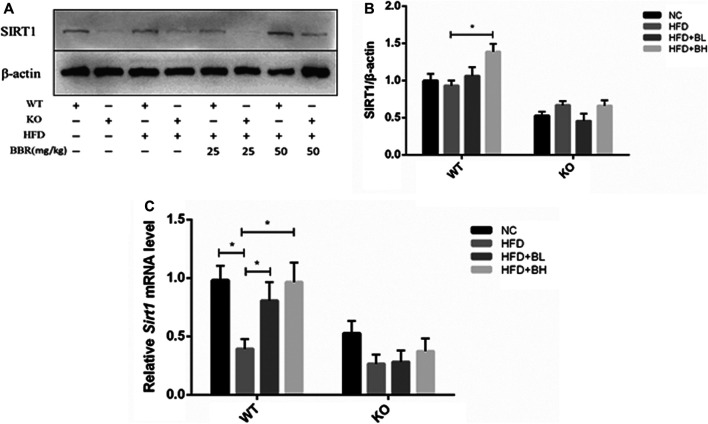
BBR upregulated Sirt1 expression in adipose tissue. Wild Type (WT) or *Sirt* ^+/−^ (KO) C57BL/6 mice fed with normal chow (NC) or high-fat diet (HFD) were treated with either 25 mg/kg (BL) or 50 mg/kg (BH) BBR for 2 weeks. **(A)** Western blot analysis of total protein extracted from epididymal adipose tissue. **(B)** Band intensities of SIRT1 were quantified by normalizing to actin (*n* = 3). **(C)** Relative mRNA levels of SIRT1 in the epididymal adipose tissue detected by real-time PCR (*n* = 3). Quantitative data are presented as mean ± SD. *p < 0.05.

**FIGURE 6:**
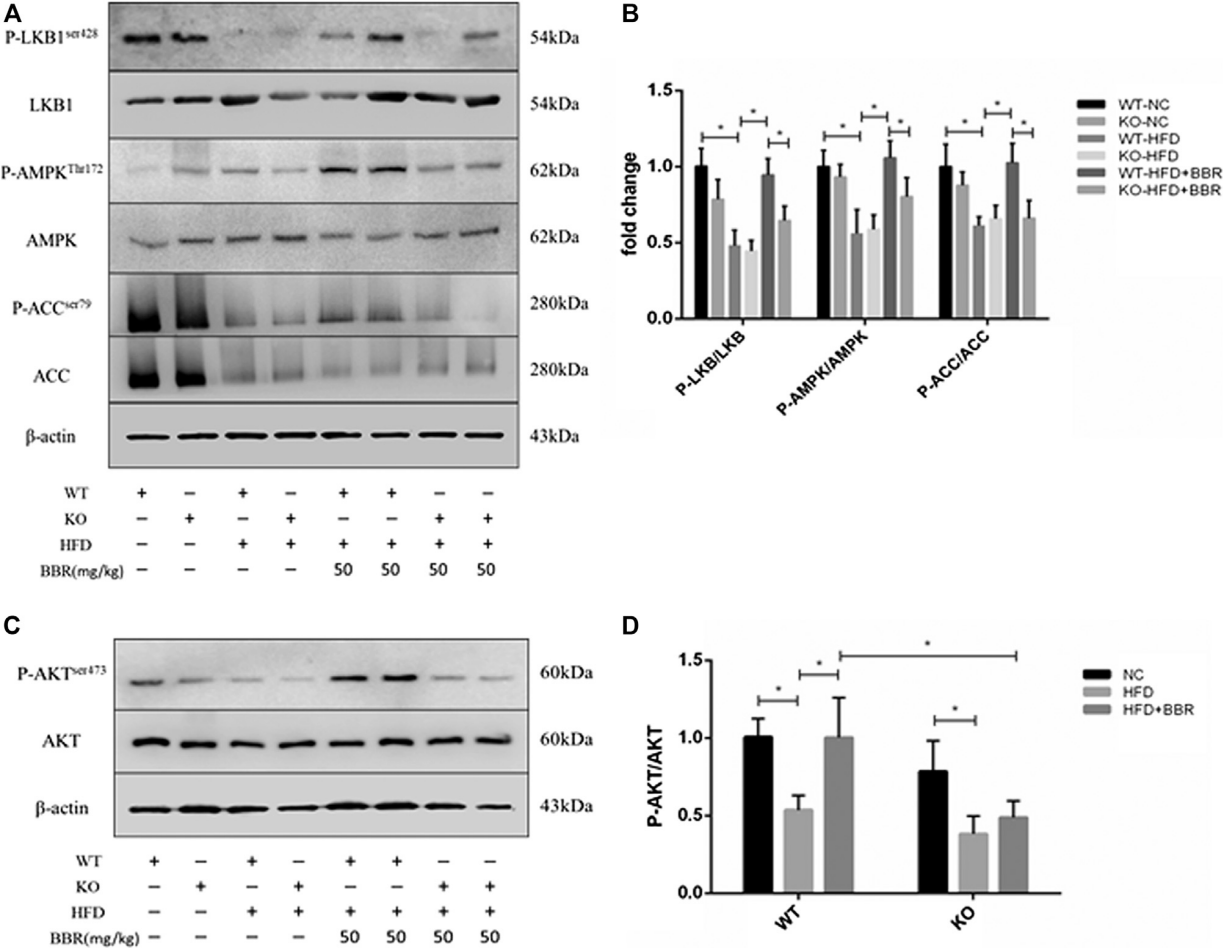
BBR-induced activation of adipose tissue AMPK and insulin pathway was regulated by SIRT1. Wild Type (WT) or *Sirt* ^+/−^ (KO) C57BL/6 mice fed normal chow (NC) or high-fat diet (HFD) were treated with 50 mg/kg (BH) BBR for 2 weeks. Total protein extracted from epididymal white adipose tissue was used for Western blot analysis to measure phosphorylation of LKB1, AMPK, ACC, and AKT. Band intensities were quantified by normalizing to the corresponding total protein levels (*n* = 3–5). Quantitative data are presented as mean ± SD. *p < 0.05.

To clarify the role of adipose tissue SIRT1 in BBR’s activation of the AMPK pathway, we first measured SIRT1 expression in epididymal fat from BBR-treated mice. In WT obese mice, BBR significantly increased *Sirt1* mRNA and protein expression compared with that in vehicle-treated obese mice (Figures 5A–C). We next measured activation of the AMPK pathway and AKT in adipose tissue. Phosphorylation of LKB1, AMPK, and ACC, as well as phosphorylation of AKT (Figures 6C, D) was significantly decreased in WT and *Sirt1* ^*+/−*^ obese mice fed with HFD. In WT obese mice, BBR treatment restored the decreased phosphorylation of LKB1, AMPK, ACC, and AKT (Figures 6A–D); however, in *Sirt1* ^*+/−*^ obese mice, BBR treatment failed to restore the degree of phosphorylation of LKB1, AMPK, ACC, and AKT (Figures 6A–D). Similar to the results seen in *vitro* experiment (Figures 2C,D and Figures 3C,D), BBR-mediated activation of the AMPK pathway and restoration of insulin signaling in adipose tissue was regulated by SIRT1.

### Berberine-Induced Suppression of Systemic and Local Inflammatory Response Was Dependent on SIRT1

**FIGURE 7:**
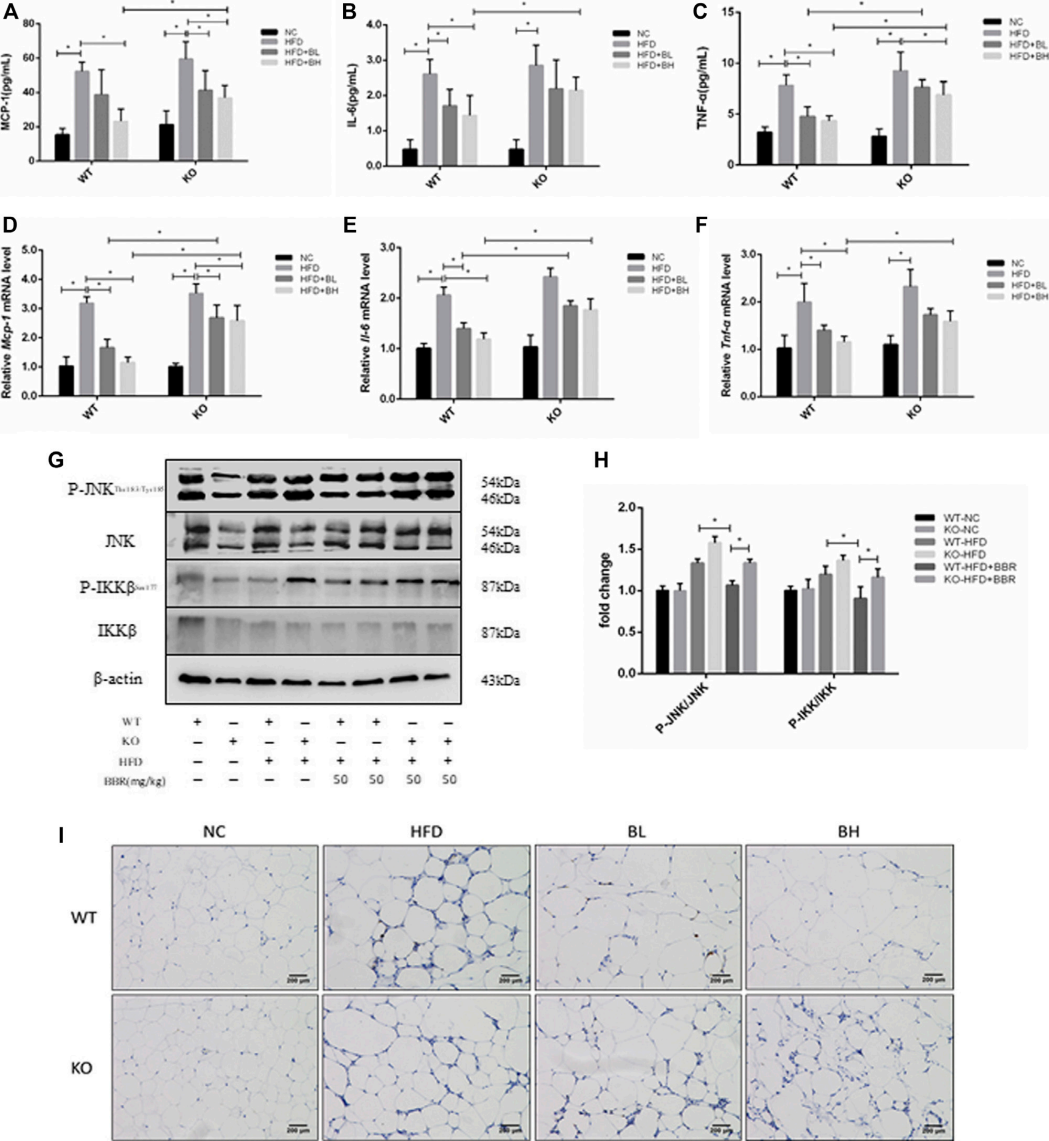
Inhibition of inflammatory response in obese mice by BBR was weakened by knockout of SIRT1. Wild Type (WT) or *Sirt* ^+/−^ (KO) C57BL/6 mice fed normal chow (NC) or high-fat diet (HFD) were treated with 50 mg/kg (BH) BBR for 2 weeks. **(A–C)** Serum inflammatory factors were measured (*n* = 6). **(B,C)** Relative expression of inflammatory factors in epididymal adipose tissue was quantified by real-time PCR (*n* = 3). **(G,H)** Phosphorylation of JNK and IKKβ in epididymal adipose tissue was measured by Western blotting. Band intensities were quantified by normalizing to the corresponding total protein levels (*n* = 3). **(I)** F4/80-positive macrophages in epididymal white adipose tissue were measured by immunohistochemistry. Quantitative data are presented as mean ± SD. *p < 0.05.

To test whether the anti-inflammatory effect of BBR was regulated by SIRT1, serum concentrations and adipose tissue expression of inflammatory cytokines, such as MCP-1, IL-6, and TNF-α, were measured in WT and *Sirt1* ^*+/−*^ mice treated with BBR. In WT obese mice, BBR significantly lowered serum levels of MCP-1, IL-6, and TNF-α in WT obese mice (*p* < 0.05; Figures 7A–C), and suppressed mRNA expression of MCP-1, IL-6, and TNF-α in adipose tissue (*p* < 0.05; Figures 7D–F). However, in *Sirt1* ^*+/−*^ obese mice treated with BBR, serum levels and expression of MCP-1, IL-6, and TNF-α in adipose tissue were significantly higher than that in WT obese mice treated with BBR (*p* < 0.05; Figures 7A–F). In addition, in WT obese mice, BBR treatment inhibited phosphorylation of JNK and IKKβ (Figures 7G,H) and reduced the immunohistochemical localization of F4/80 positive macrophages (Figure 7I) in adipose tissue. However, in the *Sirt1* ^*+/−*^ obese mice treated with BBR, JNK and IKKβ phosphorylation and the amount of F4/80 positive macrophages were significantly elevated compared with that in BBR-treated WT mice with (Figures 7G–I). These results indicated that the suppressing effects of BBR on the systemic and local inflammatory responses were mediated by SIRT1.

### Decreased Acetylation of PGC-1α by Berberine Was Restored by SIRT1 Inhibition

**FIGURE 8:**
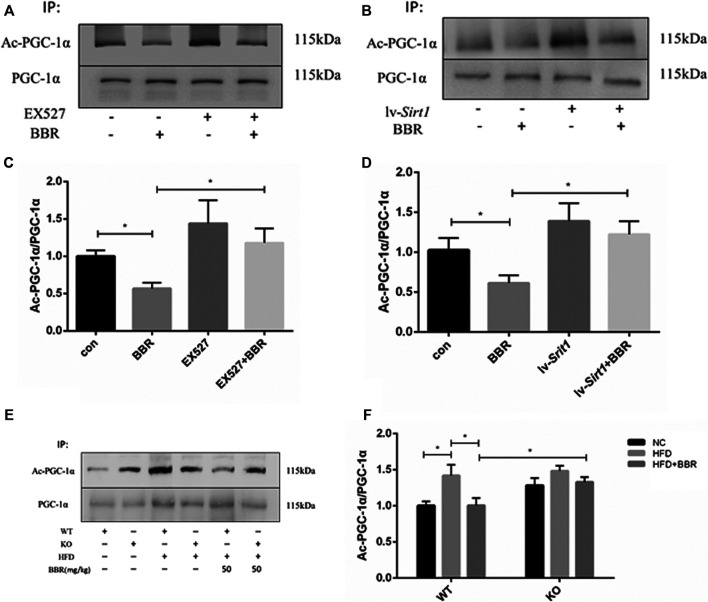
Deacetylation of PGC-1α induced by BBR was abolished by inhibition of SIRT1 **(A–D)** 3T3-L1 adipocytes were pretreated with or without 1 μmol/L EX-527, or transfected with or without 1 × 10^8^ lentiviral siRNA expression vector as described in the Materials and Methods section and then treated with 5 μM BBR for 24 h. Acetylation of PGC-1α was detected by immunoprecipitation and Western blot analysis. Band intensities were quantified by normalizing to the corresponding total protein levels (*n* = 3). **(E,F)** WT or *Sirt* ^+/−^ mice C57BL/6 fed with normal chow (NC) or high-fat diet (HFD) were treated with 50 mg/kg BBR for 2 weeks. PGC-1α acetylation in epididymal adipose tissue was measured by immunoprecipitation and Western blot analysis. Band intensities were quantified by normalizing to the corresponding total protein levels (*n* = 3). Quantitative data are presented as mean ± SD. *p < 0.05.

SIRT1 deacetylates PGC-1α (Nemoto et al., 2005; Rodgers et al., 2005); therefore, changes in PGC-1α deacetylation were evaluated after BBR treatment in adipocytes or adipose tissue. In BBR-treated 3T3-L1 adipocytes, PGC-1α acetylation was significantly reduced (Figures 8A–D). Pretreatment of adipocytes with SIRT1 inhibitor or siRNA vector led to failure of BBR treatment to suppress PGC-1α acetylation (Figures 8A–D). In agreement with the results seen in adipocytes, PGC-1α acetylation was also reduced in the adipose tissue of the WT obese mice treated with BBR, but was restored in *Sirt1* ^*+/−*^ obese mice (Figures 8E,F).

## Discussion

A number of animal and clinical studies have shown that BBR improves insulin resistance and obesity-induced metabolic dysregulation, mainly as a result of AMPK activation and inhibition of the inflammatory response in insulin-sensitive tissues (Kim et al., 2009; Chang et al., 2015; Belwal et al., 2020). In the present study, we found that BBR upregulated SIRT1 expression in adipose tissue, leading to induced activation of the AMPK pathway and suppression of obesity-related inflammatory response, resulting in improvement of local and systemic insulin resistance. Our results provide a novel insight into the molecular mechanisms involved in BBR’s action on insulin resistance and inflammation, and identify adipose tissue SIRT1 as a key regulator of the insulin sensitizing and anti-inflammatory effects of BBR.

Adipose tissue is an essential contributor to energy expenditure and also plays an important role in the development of insulin resistance and type 2 diabetes (Johnson and Olefsky, 2013). Adipose tissue SIRT1 is a key regulator of glucose homeostasis and insulin sensitivity (Cao et al., 2016). In adipocytes, SIRT1 is required for the activation of AMPK to improve insulin resistance (Price et al., 2012; Chen et al., 2018). AMPK is a central regulator of various metabolic pathways and is of therapeutic importance in the treatment of obesity, insulin resistance, type 2 diabetes, and nonalcoholic fatty liver disease (Ruderman et al., 2013). AMPK activity is reduced in adipose tissue from obese and insulin-resistant rodents and humans (Lindholm et al., 2013; Ruderman et al., 2013). BBR-induced activation of the AMPK pathway in adipocytes and adipose tissue leads to increased glucose uptake and alleviated insulin resistance (Lee et al., 2006; Yin et al., 2008). Consistent with the results of previous studies, our present study showed that BBR activated the AMPK pathway in adipocytes and adipose tissue, leading to increased glucose consumption in adipocytes and insulin sensitivity of obese mice (Lee et al., 2006; Zhou et al., 2007). SIRT1 inhibition led to abolition of BBR-induced activation of the AMPK pathway, suggesting that BBR requires SIRT1 to activate AMPK in 3T3-L1 adipocytes as well as adipose tissue. Recent studies also showed that BBR may protect various types of tissue from injury, such as cardiotoxicity (Wu et al., 2019), cognitive deficiency (Yu et al., 2018), skin wound (Zhang et al., 2020) and reperfusion injury (Lin et al., 2018), via the SIRT1 pathway. BBR was shown to increase SIRT1 expression and activate AMPK in human cervical carcinoma cells (Fusi et al., 2018). Gomes et al. reported that the BBR-induced increase in mitochondrial biogenesis and activation of AMPK induced by in muscle cells was blocked by knockdown of SIRT1 (Gomes et al., 2012). BBR has also been shown to attenuate hepatic steatosis in a SIRT1-dependent manner (Gomes et al., 2012). Our results also showed that the alleviating effect of BBR on hepatosteatosis in obese mice was to some extent weakened by SIRT1 knockout. Our *in vivo* and *in vitro* data in adipocytes and adipose tissue further our understanding of the role of SIRT1 in BBR-induced activation of the AMPK pathway.

Chronic, low-grade inflammation in adipose tissue is crucial for the development of obesity-induced insulin resistance (Makki et al., 2013; Mraz and Haluzik, 2014). Adipose tissue macrophages (ATMs) in obese mice are increased and activated, leading to the production of proinflammatory cytokines, such as TNF-α, IL-6, and MCP-1. These cytokines activate inflammatory pathways within adipocytes via paracrine mechanisms, such as JNK and IKKβ, which inhibit insulin signaling and lead to local insulin resistance. Furthermore, proinflammatory cytokines can leak out of the adipose tissue into the circulation to produce systemic insulin resistance (Johnson and Olefsky, 2013; Makki et al., 2013). BBR possesses an anti-inflammatory activity, which is responsible for its efficacy against insulin resistance (Li et al., 2014). Our previous studies showed that BBR inhibits the inflammatory response in 3T3-L1 adipocytes and adipose tissue from HFD-induced obese mice and improves insulin resistance (Shang et al., 2010; Ye et al., 2016). Recent studies have demonstrated that SIRT1 exhibits pronounced anti-inflammatory properties (Xie et al., 2013). SIRT1 has been found to suppress the inflammatory response in adipocytes, macrophages, and myeloid cells to improve insulin sensitivity (Yoshizaki et al., 2009; Yoshizaki et al., 2010; Ka et al., 2015). We found that BBR showed a reduced ability to suppress the inflammatory response in SIRT1 knockout (SIRT1^+/−^) mice, including local and systemic inflammatory response, which was indicated by the expression of TNF-α, IL-6, and MCP-1, infiltration of F4/80 positive macrophages in adipose tissue, and levels of serum TNFα, IL-6, and MCP-1. BBR’s ability to reverse the impaired insulin signaling induced by TNF-α was attenuated by inhibition or knockdown of SIRT1. Our data showed that BBR suppressed obesity-induced adipose tissue and systemic inflammation was dependent of SIRT1.

Adipose tissue comprises various cell types, including adipocytes, immune cells (macrophages and lymphocytes), preadipocytes, and endothelial cells (Olefsky and Glass, 2010; Sun et al., 2012). We previously reported that BBR suppressed LPS-induced inflammation by modulating SIRT1 in macrophages (Zhang et al., 2017). There is evidence to show that BBR also inhibits the expression of inflammatory factors and the inflammatory pathway in 3T3-L1 adipocytes (Choi et al., 2006; Ye et al., 2016). The present study used mice with non-tissue-specific knockout of SIRT1; therefore, it is not clear whether upregulation of SIRT1 by BBR in adipocytes also contributes to the suppression of inflammatory response. A recent study showed that adipocyte-specific SIRT1 knockout mice are associated with an increased number of adipose-resident macrophages and their activation, as well as increased secretion of various adipokines, including MCP-1 (Hui et al., 2017). These results indicate that SIRT1 in both adipocytes and ATMs may mediate the anti-inflammatory activity of BBR in adipose tissue. However, ATMs are thought to be a major source of proinflammatory cytokines, which decrease insulin sensitivity (Olefsky and Glass, 2010; Sun et al., 2012). In the whole body, BBR may regulate SIRT1 in ATMs by major mechanisms that suppress local and systemic obesity-induced inflammation, subsequently alleviating metabolic dysregulation.

Previous study reported that SIRT1 heterozygous knockout (*Sirt1* ^+/−^) mice fed with low fat diet does not exhibit any abnormality. But in *Sirt1* ^+/−^ mice on HFD, body weight, fat content and hepatic steatosis are increased, adipose tissue macrophage infiltration and expression of inflammatory genes, indicated by the expression of F4/80, TNF-α, IL-1, and IL-6, are enhanced in epididymal fat (Xu et al., 2010). However, in the present study, the *Sirt1* ^+/−^ obese mice induced by high fat diet showed a deterioration to some extent in metabolism-related parameters, including the level of fasting glucose and insulin, the AMPK and insulin pathway, the inflammatory response in adipose tissue, but most of which did not reach significant compared with that in the WT obese mice. The inconsistency may be due to the duration of high fat feeding, which was 12 weeks in our experiment, but 24 weeks in previous study (Xu et al., 2010). Despite this limitation, it can be concluded that adipose tissue SIRT1 play a role in the beneficial effect of BBR on obesity-related disorders from the significant different action of BBR on WT obese mice and Sirt1^+/−^ obese mice.

The AMPK pathway may link adipose tissue SIRT1 with the anti-inflammatory activity of BBR. Increased AMPK activity inhibits both LPS and palmitate-induced NF-κB signaling in macrophages (Yang et al., 2010). SIRT1 is required for the activation of AMPK and attenuates the inflammatory response in adipocytes and macrophages (Yang et al., 2010; Price et al., 2012; Chen et al., 2018). Activation of the AMPK pathway is a significant antidiabetic action of BBR (Chang et al., 2015). It is reported that BBR suppresses proinflammatory response via AMPK activation in macrophages (Jeong et al., 2009). We found that BBR activation of the AMPK pathway in adipose tissue was dependent on SIRT1, and BBR suppressed LPS-induced inflammation by modulating SIRT1 in RAW264.7 macrophages (Zhang et al., 2017). It can be concluded that the adipose tissue AMPK pathway activated by BBR-induced upregulation of SIRT1 contributes to inhibition of the inflammatory reaction, in addition to direct beneficial effects on obesity-related metabolic dysregulation. However, the alleviating effects of BBR on insulin resistance and inflammation in obese mice were not completely eliminated by SIRT1 knockout, which may be because the present study used SIRT1 heterozygous knockout mice.

SIRT1 can deacetylate various targets that regulate glucose and lipid homeostasis, including PGC-1α (Kitada and Koya, 2013). SIRT1 has been shown to directly deacetylate PGC-1α to activate its transcriptional activity (Nemoto et al., 2005; Rodgers et al., 2005). In the present study, we further demonstrated that BBR decreased PGC-1α deacetylation in adipocytes and adipose tissue, which was dependent on the SIRT1 pathway. These findings imply that SIRT1-mediated activation of PGC-1α may be another important mechanism underlying the effects of BBR on energy homeostasis. In the liver, SIRT1 deacetylates PGC-1α to activate genes involved in gluconeogenesis and fatty acid oxidation (Rodgers et al., 2008); however, the role of PGC-1α regulation by SIRT1 in adipose tissue remains unclear. Studies in adipose tissue have shown that PGC-1α may control the thermogenic program, differentiation to brown adipose tissue, triglyceride hydrolysis, and fatty acid re-esterification (Uldry et al., 2006; Mazzucotelli et al., 2007; Rius-Perez et al., 2020). Recent studies have shown that BBR enhances beige adipogenesis and increases thermogenesis in white adipocytes by recruiting PGC-1α to induce UCP1 expression. (Zhang et al., 2014; Lin et al., 2019), which may involve deacetylation of PGC-1α induced by BBR via SIRT1. The effect of BBR on the activity of PGC-1α and consequent metabolic changes require further investigated.

In summary, this is the first study to report that BBR upregulates SIRT1 expression in adipocytes and adipose tissue, leading to activation of the AMPK pathway and inhibition of the inflammatory response in adipose tissue. Our findings indicate that adipose tissue SIRT1 is a major regulator of the therapeutic effects of BBR in obesity-related insulin resistance and metabolic disorders. Our data provide a novel insight into the molecular mechanisms involved in the action of BBR on insulin resistance and inflammation.

## Data Availability Statement

The data that support the findings of this study are available from the corresponding author upon reasonable request.

## Ethics Statement

The animal study was reviewed and approved by the Animal Care and Use Committee at the University of Nanjing University of Chinese Medicine.

## Author Contributions

QY and WS designed research. YS, SZ, BG, SL, HZ, XY, JZ, and LY performed research. WS, YS, SZ, and HZ analyzed data. YS, QY, and WS wrote the paper.

## Funding

This work was supported by the National Natural Science Foundation of China (grant numbers 81873060, 81473391, and 81603569) and the Open Projects of the Discipline of Chinese Medicine of Nanjing University of Chinese Medicine Supported by the Subject of Academic priority discipline of Jiangsu Higher Education Institutions (2018-87).

## Conflict of Interest

The authors declare that the research was conducted in the absence of any commercial or financial relationships that could be construed as a potential conflict of interest.
