# Supplementary material for: Adipose Tissue SIRT1 Regulates Insulin Sensitizing and Anti-Inflammatory Effects of Berberine
Source: Front Pharmacol. 2020 Dec 17;11:591227. doi: 10.3389/fphar.2020.591227 (PMC7774030; doi:10.3389/fphar.2020.591227)
Supplement: Supplementary file 1 [file datasheet1.docx]

Supplementary Material

**S1**


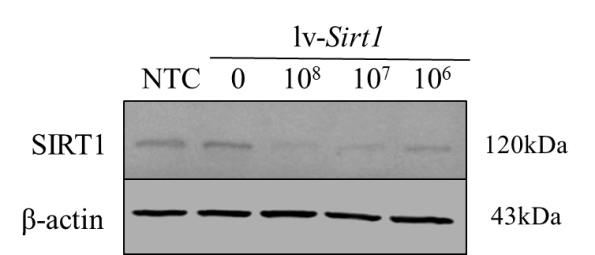


**S1. Silencing effect of different titers of lv-*Sirt1* on SIRT1 protein in 3T3-L1 cells.** Mature 3T3-LI adipocytes were infected with the lentiviral vectors at different titers for 24 h. Knockdown of Sirt1 expression was evaluated by Western blot analysis. NTC：Non-targeting control at titer of 1 × 10^8^ TU/mL

**S2**

**
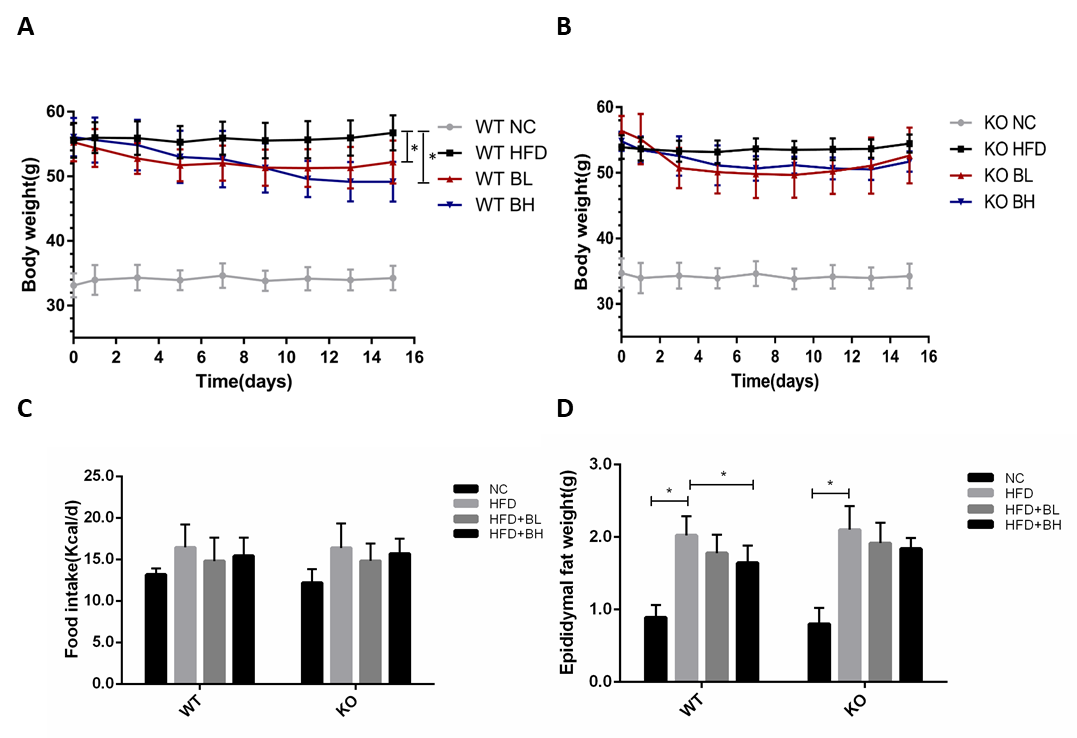
**

**S2. Effects of BBR on body weight, food intake and epididymal fat weight.** Wild Type (WT) or *Sirt*^+/−^ (KO) C57BL/6 mice fed either normal chow (NC) or high-fat diet (HFD) were treated with 25 mg/kg (BL) or 50 mg/kg (BH) BBR for two weeks. Body weight in wild type mice (A) and in KO mice (B), food intake (C) and epididymal fat pad (D) was weighted (n = 6). Data are expressed as mean ± SD. **P* < 0.05.

S3


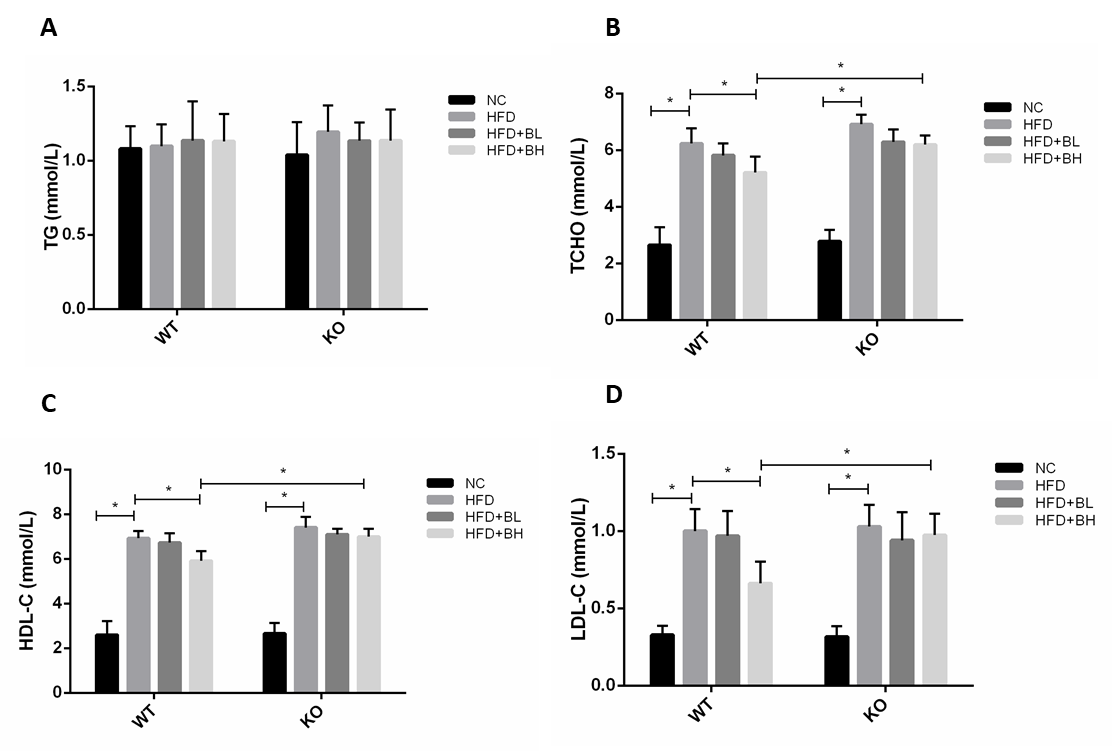


S3. **Effects of BBR on the serum lipid profile.** Wild Type (WT) or *Sirt*^+/−^ (KO) C57BL/6 mice fed either normal chow (NC) or high-fat diet (HFD) were treated with 25 mg/kg (BL) or 50 mg/kg (BH) BBR for two weeks. Serum triglyceride (TG) (A), low density lipoprotein cholesterol (LDL-C) (B)，high density lipoprotein cholesterol (LDL-C) (C) and total cholesterol (TCHO) (D) was measured.(n = 6). Data are expressed as mean ± SD. **P* < 0.05.

S4


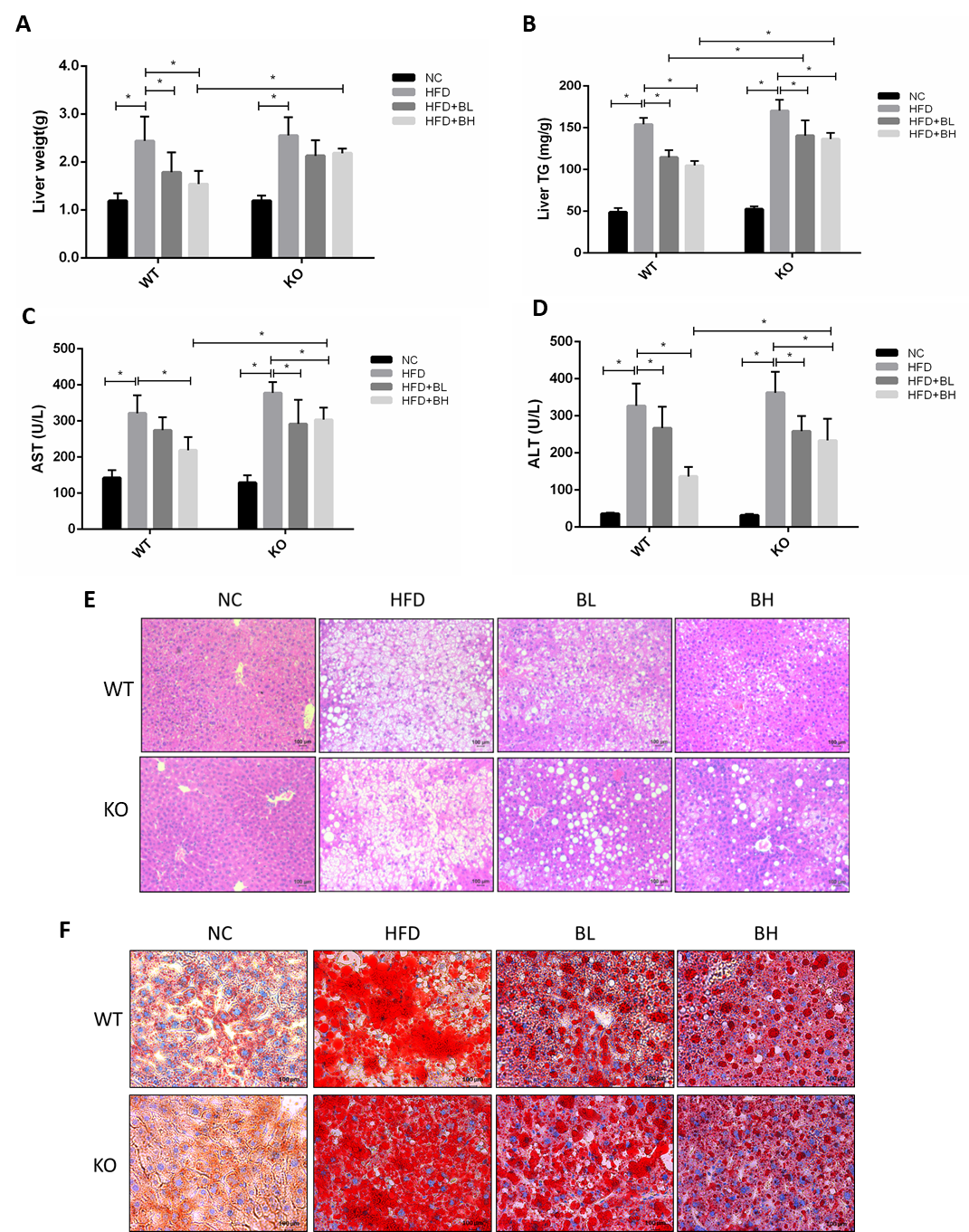


S4. **BBR-induced alleviation of hepatosteatosis was attenuated by SIRT1 knockout in HFD-induced obese mice.** Wild Type (WT) or *Sirt*^+/−^ (KO) C57BL/6 mice fed either normal chow (NC) or high-fat diet (HFD) were treated with 25 mg/kg (BL) or 50 mg/kg (BH) BBR for two weeks. Liver weight (A), content of triglyceride content in liver (B), serum aspartate transaminase (AST) (C) and alanine aminotransferase (ALT) (D) was measured (n = 6). Data are expressed as mean ± SD. **P* < 0.05. Liver sections were stained with hematoxylin and eosin (E) or oil red O (F).

**Supplemental Table 1. Sequences of the primer for Real time PCR**

| Gene | GenBank accession |  | Primer sequences |
| --- | --- | --- | --- |
| β-actin | NM_007393.5 | forward | 5′-CGTTGACATCCGTAAAGACC-3′ |
|  |  | reverse | 5′-AACAGTCCGCCTAGAAGCAC-3′ |
| SIRT1 | NM_001159589.2 | forward | 5′-GTCATAGGCTAGGTGGTGAAT-3′ |
|  |  | reverse | 5′-GTTGGTGGCAACTCTGATAAA-3′ |
| SIRT2 | NM_001122765.1 | forward | 5′-CAGCTACTTCAAGAAACATCCG-3′ |
|  |  | reverse | 5′-TATTCTTTTCTGCAGGAGGTGT-3′ |
| SIRT3 | NM_001127351.1 | forward | 5′-TCTATACACAGAACATCGACGG-3′ |
|  |  | reverse | 5′-GCATGTAGCTGTTACAAAGGTC-3′ |
| SIRT4 | NM_001167691.1 | forward | 5′-CATCCAGCACATTGATTTCGTC-3′ |
|  |  | reverse | 5′-GTTGGTGAGAGGAGAATTGAGG-3′ |
| SIRT5 | NM_178848.3 | forward | 5′-CGATTCATTTCCCAGTTGTGTT-3′ |
|  |  | reverse | 5′-CATATTTGAACTTGGACGAGCC-3′ |
| SIRT6 | NM_001163430.1 | forward | 5′-CCCAAGTGTAAGACGCAGTA-3′ |
|  |  | reverse | 5′-GTCCAGAATGGTGTCTCTCAG-3′ |
| SIRT7 | NM_001363439.1 | forward | 5′-GGTCCAGCTTGAAGGTACTAAA-3′ |
|  |  | reverse | 5′-CAGGTTCACGATGTAGAGTTTG-3′ |
| TNF-α | NM_001278601.1 | forward | 5′-ACGGCATGGATCTCAAAGAC-3′ |
|  |  | reverse | 5′-GTGGGTGAGGAGCACGTAGT-3′ |
| MCP-1 | NM_011333.3 | forward | 5′-CCACAACCACCTCAAGCACT-3′ |
|  |  | reverse | 5′-TAAGGCATCACAGTCCGAGTC-3′ |
| IL-6 | NM_001314054.1 | forward | 5′-AGTTGCCTTCTTGGGACTGA-3′ |
|  |  | reverse | 5′-AAGGGAATACCATAACATCA-3′ |
